# tRF‐34‐P4R8YP9LON4VHM Promotes Hepatocellular Carcinoma Progression and Tumour Cell‐Induced Angiogenesis via the MEK/ERK Pathway

**DOI:** 10.1111/jcmm.70560

**Published:** 2025-04-22

**Authors:** Tianxin Xu, Han Hua, Fei Song, Nannan Zhang, Cheng Gao, Zhong Chen

**Affiliations:** ^1^ Department of General Surgery Affiliated Hospital of Nantong University, Medical School of Nantong University Nantong China; ^2^ Department of Anesthesiology and Surgery Affiliated Hospital of Nantong University, Medical School of Nantong University Nantong China

**Keywords:** angiogenesis, hepatocellular carcinoma, metastasis, tRNA‐derived small RNAs, tumour growth

## Abstract

Hepatocellular carcinoma (HCC) is the most common form of primary liver cancer and poses a significant global health challenge. In recent years, tRNA‐derived small RNAs (tsRNAs) have gained significant attention due to their potential role in various cancers, including HCC. In this study, we reported that tRF‐34‐P4R8YP9LON4VHM expression was elevated in HCC tissues and cell lines. The association between tRF‐34‐P4R8YP9LON4VHM expression and HCC patients' clinicopathological parameters was determined using tissue microarrays of 90 patients, and we found that it was positively associated with the level of AFP, tumour size, microvascular density (MVD), and TNM stage. We performed CCK8, colony formation assay, EdU, cell cycle analysis, transwell assay, and tube formation assay to verify that tRF‐34‐P4R8YP9LON4VHM could enhance HCC proliferation, migration, invasion, and tumour cell‐induced angiogenesis in vitro and in vivo. Mechanistically, tRF‐34‐P4R8YP9LON4VHM could downregulate DAB2IP expression by directly targeting its 3'‐UTR, consequently activating the MEK/ERK signalling pathway and promoting the secretion of VEGFA from HCC cells into the supernatant. In conclusion, our research indicated that tRF‐34‐P4R8YP9LON4VHM might act as a crucial player in molecular mechanisms and provide novel treatment strategies for HCC patients.

## 1 Introduction

Hepatocellular carcinoma (HCC) is the third most common cause of cancer‐related deaths and accounts for 75%–85% of primary liver cancers [1]. The biological process of HCC is complex, involving multiple factors that disrupt the balance between the inactivation of tumour suppressors and activation of oncogenic molecules, leading to abnormal activation of molecular signalling pathways, cell differentiation, and dysregulation of angiogenesis [2]. HCC is a solid tumour characterised by a high degree of capillarisation and arterialization [3]. Due to the crucial role in tumour growth and metastasis, angiogenesis was considered a significant predictor of overall survival in HCC patients [4]. Although some anti‐angiogenic drugs were widely used in the treatment of HCC, the outcomes remained poor [5]. Hence, exploration of the molecular mechanisms and identification of a novel therapeutic target for angiogenesis to improve HCC prognosis are urgently needed.

In recent years, the increased use of high‐throughput sequencing technologies has led to the identification of a growing number of non‐coding RNA types [6, 7]. A recently identified group of non‐coding RNAs was transfer RNA‐derived small RNAs (tsRNAs), which included tRNA‐derived stress‐induced RNAs (tiRNAs) and tRNA‐derived RNA fragments (tRFs) [8, 9]. Mechanically, It has been suggested that tsRNAs can interact with messenger RNA (mRNA) or RNA‐binding proteins to modulate the stability and translation of target mRNAs based on the localization of tsRNAs [10]. Further studies have revealed that tsRNAs could influence translation by modulating the initiation, elongation, and ribosome genesis process, thereby affecting protein production [11, 12, 13]. Meanwhile, growing evidence indicated the correlation between dysregulation of tsRNAs and various angiogenesis‐related diseases [14]. For instance, Jiang et al. [15] discovered that tRF‐1001 suppressed the expression of RBPJ and MAML1 by modulating METTL3‐mediated m^6^A modification, ultimately inhibiting endothelial cell sprouting and pathological angiogenesis. Liu et al. [16] also found that the expression of tRF‐22‐8BWS72092 was significantly downregulated in the choroidal tissue of myopic patients, highlighting its protective role in regulating choroidal vasculopathy. In vitro, tRF‐22‐8BWS72092 exhibited anti‐angiogenic effects, reducing the proliferation, migration, and tube formation abilities of choroidal endothelial cells, which contributed to controlling myopia progression. In addition, Liang et al. [17] demonstrated that tRF‐Glu‐CTC regulates angiogenesis by silencing the expression of VASH1 and promoting the secretion of inflammatory factors, thereby accelerating the progression of neovascular age‐related macular degeneration (AMD). Although these three studies primarily focused on ocular angiogenic diseases, their findings suggested that tsRNAs might also hold potential therapeutic implications for other types of angiogenic diseases, including cancer.

In this study, we reported that tRF‐34‐P4R8YP9LON4VHM, a 5'‐tiRNA‐Gly‐GCC that was elevated in HCC tissues, could silence the expression of DAB2IP by directly targeting its 3'‐UTR. Subsequently, tRF‐34‐P4R8YP9LON4VHM could enhance HCC proliferation, migration, invasion, and tumour cell‐induced angiogenesis by activating the MEK/ERK signalling pathway and promoting the secretion of VEGFA. These findings offered deeper insights into the role of tRF‐34‐P4R8YP9LON4VHM in HCC tumorigenesis, illustrating its potential as a new target for anti‐angiogenic cancer treatment.

## 2 Materials and Methods

### 2.1 Patients and Tissue Specimens

45 paired HCC tissues and their adjacent normal tissues (NAT) that were undergoing routine surgery were collected from the Affiliated Hospital of Nantong University (Nantong, Jiangsu, China). In another cohort, 90 paired HCC specimens were treated with formalin paraffin‐embedded tissue to prepare tissue chips for IHC and ISH. None of the patients had received systematic therapy before surgery. Informed written consent was obtained from each patient participating in this study. The study protocol was approved by the Ethics Committee of the Affiliated Hospital of Nantong University (ethical review report number: 2022‐L062). All investigations were conducted in accordance with the principles outlined in the 1975 Declaration of Helsinki.

### 2.2 Cell Lines and Culture

HCC cell lines (HepG2, Hep3B, Huh7, MHCC97H, HCCLM3, and PCL/PRF/5) were sourced from SIBCB (Shanghai Institute of Biochemistry and Cell Biology, Shanghai, China). HepG2, Huh7, MHCC97H, HCCLM3, and PCL/PRF/5 cells were cultured in high‐glucose DMEM (GIBCO), while Hep3B cells were grown in MEM (Invitrogen) supplemented with GlutaMAX, nonessential amino acids (NEAA), and sodium pyruvate. All media were supplemented with 10% fetal bovine serum (FBS) (GIBCO) and 1% penicillin–streptomycin (Pen/Strep) mixture (HyClone, Logan, UT, USA) and incubated at 37°C with 5% CO_2_.

### 2.3 Construction of Stable Cell Lines

To obtain cell lines, stably overexpressing DAB2IP, HCCLM3, and PLC/PRF/5 cells in a good condition and logarithmic growth phase were infected with Lv‐DAB2IP and Lv‐control viruses (GenePharma, Shanghai, China). For further analysis, cell RNA or protein was extracted and the infection efficiency was confirmed by qRT‐PCR and western Blot.

### 2.4 RNA Extraction, cDNA Synthesis, and Real‐Time RT‐PCR

Total RNA from tissue and cell samples was extracted using the TRIzol RNA Extraction Kit (Invitrogen, Carlsbad, CA, USA). RNA concentration and purity were assessed by measuring the OD value at 260 nm and 280 nm, with the OD 260/280 ratio falling between 1.8 and 2.1. cDNA was amplified by the Revert Aid RT Reverse Transcription Kit (Thermo Fisher Scientific, USA) in a total of 10 μL. qRT‐PCR was conducted using the FastStart Universal SYBR Green Master Mix (Roche, Mannheim, Germany) on a QuantStudio 5 system (Thermo, Waltham, MA, USA) in the reaction mixture of 20 μL. RNU6B (U6) was used as an internal control to normalise the relative expression of tRF‐34‐P4R8YP9LON4VHM. The $2^{-\Delta \Delta C_{t}}$ method was employed to analyse the relative expression level data [18]. The ΔΔ*C*_t_ value was calculated as the difference between the experimental group (*C*_t_^target^—*C*_t_^reference^) and the control group (*C*_t_^target^—*C*_t_^reference^).

### 2.5 Nuclear‐Cytoplasmic Fraction Analysis

The cellular localisation of tRF‐34‐P4R8YP9LON4VHM was evaluated by nuclear–cytoplasmic separation assay. Nuclear and cytoplasmic RNA were isolated from the HCCLM3 and PLC/PRF/5 cell lines using the PARIST kit, following the manufacturer's protocol (Invitrogen, Thermo Fisher Scientific, USA). The RNA expression of tRF‐34‐P4R8YP9LON4VHM in both the nucleus and cytoplasm was measured using qRT‐PCR. U6 and GAPDH were used as nuclear and cytoplasmic references, respectively.

### 2.6 IHC, ISH, and FISH

IHC assays were performed with anti‐Ki‐67 antibody, anti‐E‐cadherin antibody, anti‐N‐cadherin antibody, anti‐Vimentin antibody, anti‐VEGFA antibody, and anti‐CD34 antibody. The ISH and FISH probe used for detecting tRF‐34‐P4R8YP9LON4VHM‐labelled digoxin was designed and synthesised by Servicebio Technology Co. Ltd. (Wuhan, China) in accordance with the manufacturer's instructions. The staining intensity was scored as follows: 0 (no staining), 1 (weak), 2 (moderate), and 3 (strong). The staining extent was scored as follows: 0 (< 10%), 1 (11%–25%), 2 (26%–50%), 3 (51%–75%), and 4 (76%–100%). The final tRF‐34‐P4R8YP9LON4VHM expression score was calculated by multiplying the intensity score by the extent score, yielding a range from 0 to 12. Sections with a total score of 6 or higher were classified as the high expression group, whereas those with a score lower than 6 were assigned to the low expression group. The IHC and ISH scores were assessed by two pathologists in a two‐blinded manner.

### 2.7 Dual‐Luciferase Reporter Assay

As DAB2IP was the target of tRF‐34‐P4R8YP9LON4VHM, we used the mutant (MUT) and wild type (WT) of the DAB2IP 3′ UTR to treat PmirGLO vectors (Promega, Madison, WI, USA). The recombined PmirGLO vectors were then co‐transfected with the tRF‐34‐P4R8YP9LON4VHM mimics or Mimics‐NC into HCCLM3 and PLC/PRF/5 cells to detect the relative luciferase activity.

### 2.8 CCK8

For the CCK8 assay, treated cells were seeded into 96‐well plates at a concentration of approximately 3 × 103 cells per well and incubated for 1–5 days. Each well was then cultured with 100 μL of complete growth medium, which contained 10% FBS. Subsequently, 10 μL of the CCK‐8 detection solution (Catalogue Number: CK04, Dojindo Laboratories, Japan) was introduced into each well, followed by a 2 h incubation period. The absorbance at 450 nm (OD450) was subsequently determined using a microplate reader (TECAN‐Spark, Switzerland).

### 2.9 Colony Formation Assay

For the colony formation assay, 1 × 10^3^ cells were seeded in 2 mL of complete culture medium in a 6‐well plate and cultured for 14 days. After 2 weeks, colonies were fixed with a 4% paraformaldehyde solution and stained with crystal violet. The number of cell clones was quantified using ImageJ software v1.47 (NIH, USA) 19.

### 2.10 EdU

The CellorLab EdU Cell Proliferation Kit with Alexa Fluor 555 (Epizyme, Shanghai, China) was utilised to measure the proportion of proliferating cells. According to the manufacturer's instructions, approximately 5 × 105 cells in 6‐well plates were cultured in complete medium with a concentration of 10 μM EdU and incubated for 2 h at 37°C with 5% CO_2_. After labelling, cells in each well were initially fixed, permeabilised, and stained with Alexa Fluor 555 under dark conditions. After staining with Alexa Fluor 555, the cells were further stained with DAPI for 10 min to visualise the cell nuclei. Finally, the cells were examined via fluorescence microscopy (Olympus, Tokyo, Japan).

### 2.11 Cell Cycle Analysis

Flow cytometry was used to assess the cell cycle. At 48 h after transfection, the cell percentage was stained using a PI‐based cell cycle assay kit (Invitrogen). The percentage of the cells in the G1, S, and G2 phases was analysed using Flowjo 10.8. Each assay was repeated three times.

### 2.12 Transwell Assay

For the migration assay, cells were digested with trypsin–EDTA 48 h after transfection. The transfected cell suspension was then seeded into the upper chamber of Transwell inserts (Corning Inc., Costar, USA). The upper chamber was added with 100 μL of serum‐free medium containing 5 × 10^4^ cells, while 500 μL of medium containing 10% (v/v) FBS was added to the lower chamber. The cells were incubated at 37°C with 5% CO_2_ for 48 h. The non‐migrated cells in the upper chamber were removed using a cotton‐tipped applicator, and the cells on the lower surface of the inserts were fixed and stained with 0.2% crystal violet. As for cell invasion capability, the upper chamber of the Transwell inserts was treated with Matrigel, and the remaining steps were the same as those in the Transwell migration assay. The number of migrated cells on the underside of the inserts was quantified using ImageJ software v1.47.

### 2.13 Tube Formation Assay

For tube formation assay, 100 μL of Matrigel (BD Biosciences, San Jose, CA, USA) was added to each well of a 48‐well plate. After polymerising at 37°C for 30 min, HUVECs were seeded onto the Matrigel at the density of approximately 50,000 cells per well and incubated with conditioned medium collected from HCC cells transfected with tRF‐34‐Mimics, Mimics‐NC, tRF‐34‐Inhibitor, and Inhibitor‐NC. After being incubated for 6 h at 37°C with 5% CO_2_, tubules were visualised under an inverted microscope.

### 2.14 Western Blot Assays

After loading the sample, the protein was completely separated by constant current electrophoresis. After the protein was transferred to the polyvinylidene fluoride (PVDF) membrane, it was blocked with 5% skim milk or 5% BSA for 1 h at room temperature and incubated with primary antibodies overnight at 4°C. Primary antibodies were generated against DAB2IP (diluted 1:1000, #ab87811, Abcam, Cambridge, UK), E‐cadherin (1:2000, #60335‐1‐Ig, Proteintech), N‐cadherin (1/4000, #66219‐1‐Ig, Proteintech), Vimentin (1:20,000, #60330‐1‐AP, Proteintech), MEK1/2 (1:5000, #11049‐1‐AP, Proteintech), phospho‐MEK1/2 (1:1000, #9154, Cell Signalling Technology), ERK1/2 (1:2000, #66192‐1‐Ig, Proteintech), phospho‐ERK1/2 (1:1000, #28733‐1‐AP, Proteintech), and VEGFA (1:1000, #66828‐1‐Ig, Proteintech), and GAPDH (1:50,000, #60004‐1‐Ig, Proteintech) was used as an internal control. On the second day, secondary antibodies specific to the primary antibodies were added. Photos were taken with the ChemiDoc Imaging System (Bio‐Rad, USA).

### 2.15 Xenograft Tumours In Vivo

For nude mouse tumour formation experiments, female BALB/c nude mice (5 weeks old, 18–21 g body weight) were purchased from the Laboratory Animal Center of Nantong University. All mice were approved by the Animal Experimental Ethical Committee of Nantong University (ethical review report number: S20220228‐004) and kept under specific pathogen‐free conditions. PLC/PRF/5 cells were transfected with tRF‐34‐antagomir and NC‐antagomir. Each group was resuspended in serum‐free DMEM at a density of 50,000,000 cells per mL, and then, 0.1 mL of the suspension was subcutaneously injected into the left back of the nude mice. Tissues were embedded, sliced, and stained with HE and IHC for further analysis.

### 2.16 Statistical Analysis

All data were presented as the mean ± standard deviation (SD) and analysed with SPSS 24.0 statistical software (IBM SPSS Statistics, Chicago, USA) or GraphPad PRISM 8.0 (GraphPad Software, San Diego, CA, USA). Pearson's chi‐squared test was utilised to evaluate the correlation between tRF‐34‐P4R8YP9LON4VHM and clinicopathological factors in HCC. Student's *t*‐test or one‐way ANOVA were utilised to analyse the differences between experimental groups. For the analysis of survival data, Kaplan–Meier curves were constructed, and the log‐rank test was performed. Statistical significance was set at * *p* < 0.05, ** *p* < 0.01, and *** *p* < 0.001. Differences were considered statistically significant at *p* < 0.05. All experiments were performed in triplicate to ensure result reliability.

## 3 Results

### 3.1 Upregulation of tRF‐34‐P4R8YP9LON4VHM in HCC Tissues and Cell Lines

**FIGURE 1:**
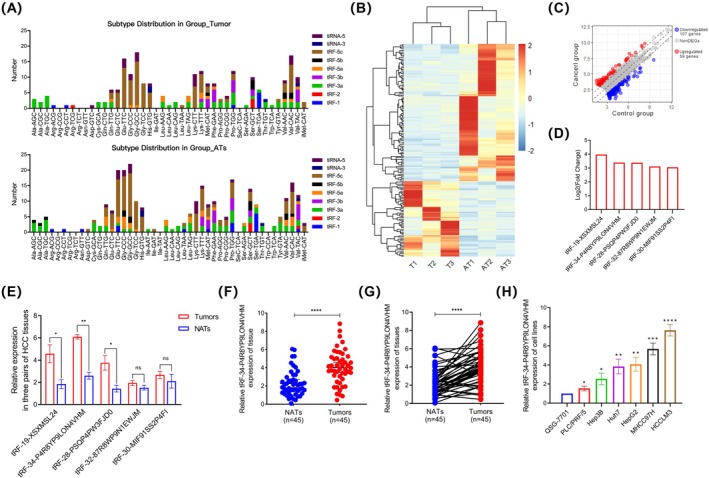
The expression of tRF‐34‐P4R8YP9LON4VHM was upregulated in HCC tissues and cell lines. (A) The stacked bar chart of tumours and normal‐adjacent tissues. (B) Heatmap displaying differentially expressed tsRNAs between three pairs of HCC tissues and NATs. (C) Scatter plots of upregulated and downregulated tsRNAs. (D) Top 5 upregulated tsRNAs in tumour tissues. (E) The relative expression levels of top 5 upregulated tsRNAs in 3 pairs of HCC tissues. (F, G) Unpaired scatter plot and paired line plot showed the high expression of tRF‐34‐P4R8YP9LON4VHM in HCC tissues and NATs. (H) Relative expression of tRF‐34‐P4R8YP9LON4VHM in different HCC cell lines. All data are shown as the mean ± SEM of 3 independent experiments. *p < 0.05; **p < 0.01; ***p < 0.001.

To explore the expression differences of tsRNAs in HCC, we first used high‐throughput sequencing technology to compare tsRNAs expression between tumours and normal‐adjacent tissues (NATs) from three pairs of HCC patients. The same tRNA or pre‐tRNA can be specifically cleaved at different cutting sites by specific enzymes, producing various tsRNAs. Therefore, we classified them into different subtypes according to their different tRNA or pre‐tRNA sources. The stacked bar chart shows that in cancer tissues, the most abundant subtypes of tRNAs come from Gly‐GCC, Gly‐CCC, and Glu‐TTC, while in NATs, the most abundant subtypes come from Gly‐GCC, Val‐CAC, and Glu‐TTC, with tRF‐5c and 5'‐tiRNA accounting for the largest proportion among these subtypes (Figure 1A). Heatmap and scatter plot showed that there were 166 significantly differentially expressed tsRNAs (*p* < 0.05 and |Log_2_FC| > 2.5), of which 59 tsRNAs were upregulated and 107 were downregulated (Figure 1B,C). The top 5 upregulated tsRNAs were shown in Figure 1D. To screen for potential carcinogenic tsRNAs, the top 5 tsRNAs with the greatest differences were selected as candidates and were examined in an additional 3 pairs of HCC tissues. Notably, the qRT‐PCR results showed that the expression of tRF‐34‐P4R8YP9LON4VHM was significantly higher in tumours than in NATs (Figure 1E). This finding was further verified using qRT‐PCR in 45 pairs of HCC tissues. Unpaired scatter plot and paired line plot showed that the expression of tRF‐34‐P4R8YP9LON4VHM was significantly upregulated in HCC tissues (Figure 1F,G). Furthermore, the expression of tRF‐34‐P4R8YP9LON4VHM was detected in different HCC cell lines (PLC/PRF/5, Hep3B, Huh7, HepG2, MHCC97H, and HCCLM3), and we found that tRF‐34‐P4R8YP9LON4VHM was significantly increased in HCC cell lines as compared to human hepatocytes (Figure 1H). Considering the absence of dedicated studies on this novel tsRNA, our investigation focused on tRF‐34‐P4R8YP9LON4VHM, which was abbreviated to tRF‐34‐P4R, as a pivotal molecule that might be substantially involved in the development of HCC.

### 3.2 Brief Introduction of tRF‐34‐P4R8YP9LON4VHM

tsRNAs are categorised into two main types: tiRNAs, which are tRNA halves (5′‐tiRNA and 3′‐tiRNA), and tRFs (1‐tRF, 3‐tRF, 5‐tRF and i‐tRF). According to the UCSC Genome Browser database, tRF‐34‐P4R8YP9LON4VHM is located on chromosome chr1 (q23.3) with coordinates of 161,413,094 to 161,413,127 (Figure S1A). In the MINTbase v2.0 database, tRF‐34‐P4R8YP9LON4VHM has a length of 34 nt and is the 5′‐half of the mature tRNA‐Gly‐GCC (Figure S1B). The cleavage sites are indicated with red scissors symbols according to the online database tRNAdb (Figure S1C). In addition, sanger sequencing of the qRT‐PCR product confirmed that the product contained the full‐length sequence of tRF‐34‐P4R8YP9LON4VHM (5′‐GCATGGGTGGTTCAGTGGTAGAATTCTCGCCTGC‐3′) (Figure S1D). Finally, to explore the intracellular localisation of tRF‐34‐P4R8YP9LON4VHM in HCC cell lines, a nuclear‐cytoplasmic separation assay was utilised to demonstrate that tRF‐34‐P4R8YP9LON4VHM was mainly located in the cytoplasm in the indicated cell lines (Figure S1E).

### 3.3 tRF‐34‐P4R8YP9LON4VHM Overexpression Indicates Poor Prognosis in HCC

**FIGURE 2:**
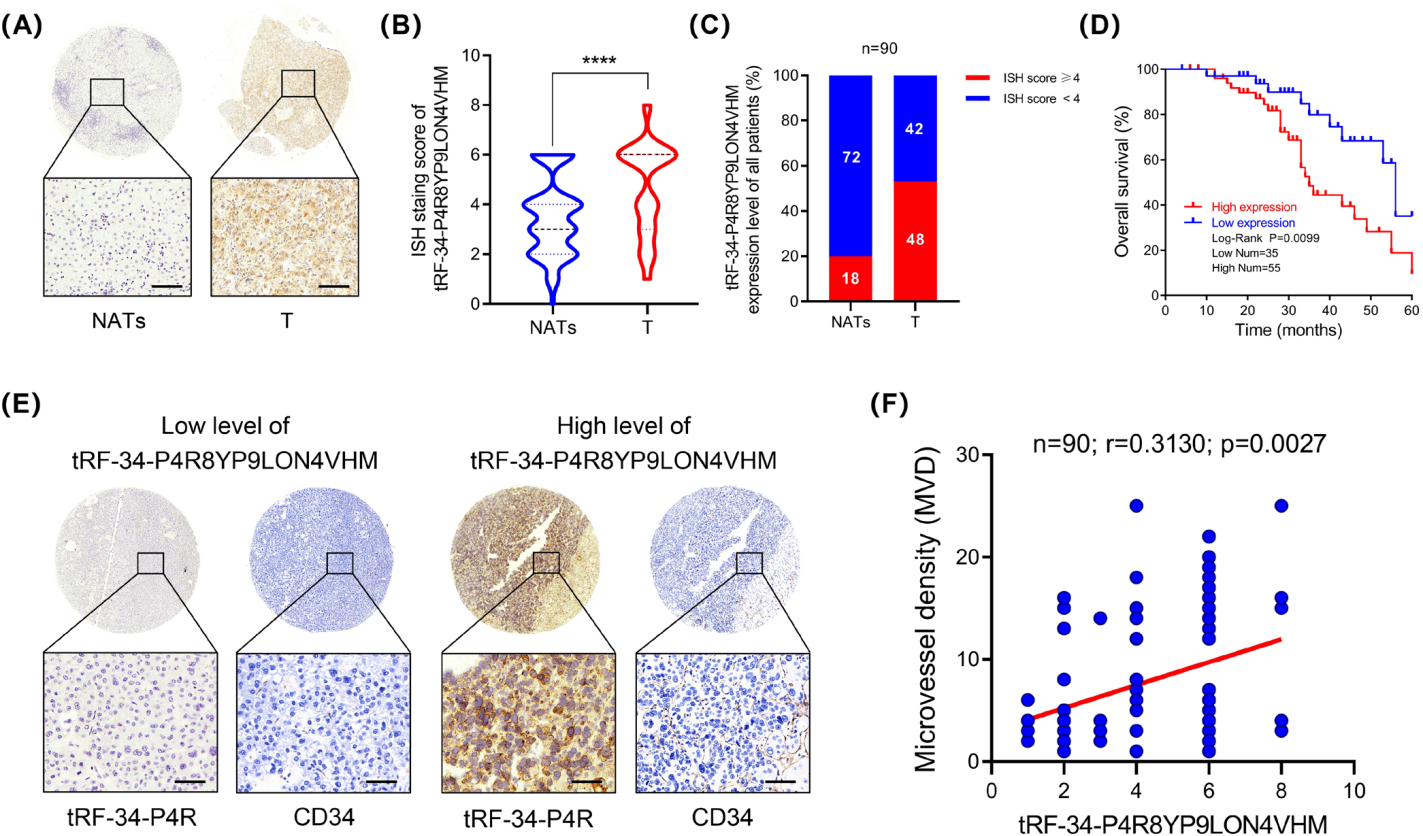
Clinicopathological analysis of tRF‐34‐P4R8YP9LON4VHM. (A) Representative images of ISH staining for tRF‐34‐P4R8YP9LON4VHM expression in HCC tissues and NATs. Scale bar, 100 μm. (B) ISH staining score of tRF‐34‐P4R8YP9LON4VHM in HCC tissue microarrays. (C) The percentages of high & low cases in HCC tissues and NATs. (D) Kaplan–Meier curves of tRF‐34‐P4R8YP9LON4VHM expression in HCC patients (*n* = 90). (E) Representative images of IHC staining for CD34 expression among tRF‐34‐P4R8YP9LON4VHM‐high patients and tRF‐34‐P4R8YP9LON4VHM‐low patients. (F) Correlation analysis between the expression of tRF‐34‐P4R8YP9LON4VHM and microvascular density (MVD). Scale bar, 100 μm. All data are shown as the mean ± SEM of 3 independent experiments. *p < 0.05; **p < 0.01; ***p < 0.001.

To further investigate the clinical significance of tRF‐34‐P4R8YP9LON4VHM in HCC, we conducted ISH experiments to assess its expression in HCC tissue microarrays of 90 patients. The staining score for tRF‐34‐P4R8YP9LON4VHM in HCC tissue was significantly higher in tumour tissues than that in NATs (Figure 2A–C). Further analysis revealed that among 90 HCC patients, 55 patients had a higher staining score for tRF‐34‐P4R8YP9LON4VHM in tumour tissues compared to NATs, while in the remaining 35 patients, the staining score for tRF‐34‐P4R8YP9LON4VHM in tumour tissues was lower. Based on this result, we categorised the 90 HCC patients into the tRF‐34‐P4R8YP9LON4VHM high‐expression group (*n* = 55) and tRF‐34‐P4R8YP9LON4VHM low‐expression group (*n* = 35) for subsequent clinicopathological analysis. The results indicated that high expression of tRF‐34‐P4R8YP9LON4VHM was associated with high level AFP, long tumour diameter, and TNM stage but showed no significant association with age, gender, HBsAg, Child‐Pugh classification, liver cirrhosis, and portal vein thrombosis (Table 1). Kaplan–Meier survival curve analysis indicated that HCC patients with high expression of tRF‐34‐P4R8YP9LON4VHM had shorter overall survival (Figure 2D). Since tumour angiogenesis is a key factor in the growth and metastasis of HCC, we proceeded to examine the expression of the endothelial cell surface marker CD34 in tissue microarrays. IHC results showed that the number of CD34‐positive patients was larger in those with high expression of tRF‐34‐P4R8YP9LON4VHM compared to those with low expression (Figure 2E). Correlation analysis demonstrated a significant association between high expression of tRF‐34‐P4R8YP9LON4VHM and high microvascular density (MVD) (Figure 2F).

**TABLE 1:** The association between tRF‐34‐P4R8YP9LON4VHM expression and clinicopathologic parameters in tissue microarrays of 90 HCC patients.

Characteristics	Group	tRF‐34‐P4R8YP9LON4VHM		Total (*n* = 90)	*p*
		Low expression (*n* = 35)	High expression (*n* = 55)		
Age	< 60	11	25	36	0.185
	≥ 60	24	30	54	
Gender	Male	19	35	54	0.377
	Female	16	20	36	
HbsAg	Negative	11	15	26	0.672
	Positive	24	40	64	
Cirrhosis	Negative	11	23	34	0.322
	Positive	24	32	56	
Child‐Pugh classification	A	29	41	70	0.355
	B or C	6	14	20	
AFP (ng/mL)	< 20	25	24	49	0.010*
	≥ 20	10	31	41	
Diameter	≤ 5 cm	24	23	47	0.013*
	> 5 cm	11	32	43	
Portal vein thrombus	Negative	1	8	9	0.072
	Positive	34	47	81	
TNM stage	Ia stage	22	16	38	0.004**
	Ib stage	10	23	33	
	II–III stage	3	16	19	

*Note:* *p < 0.05; **p < 0.01.

Abbreviation: AFP, Alpha‐Fetoprotein.

### 3.4 tRF‐34‐P4R8YP9LON4VHM Promotes Cell Growth, Migration, and Invasion in HCC Cells and Drives Angiogenesis

**FIGURE 3:**
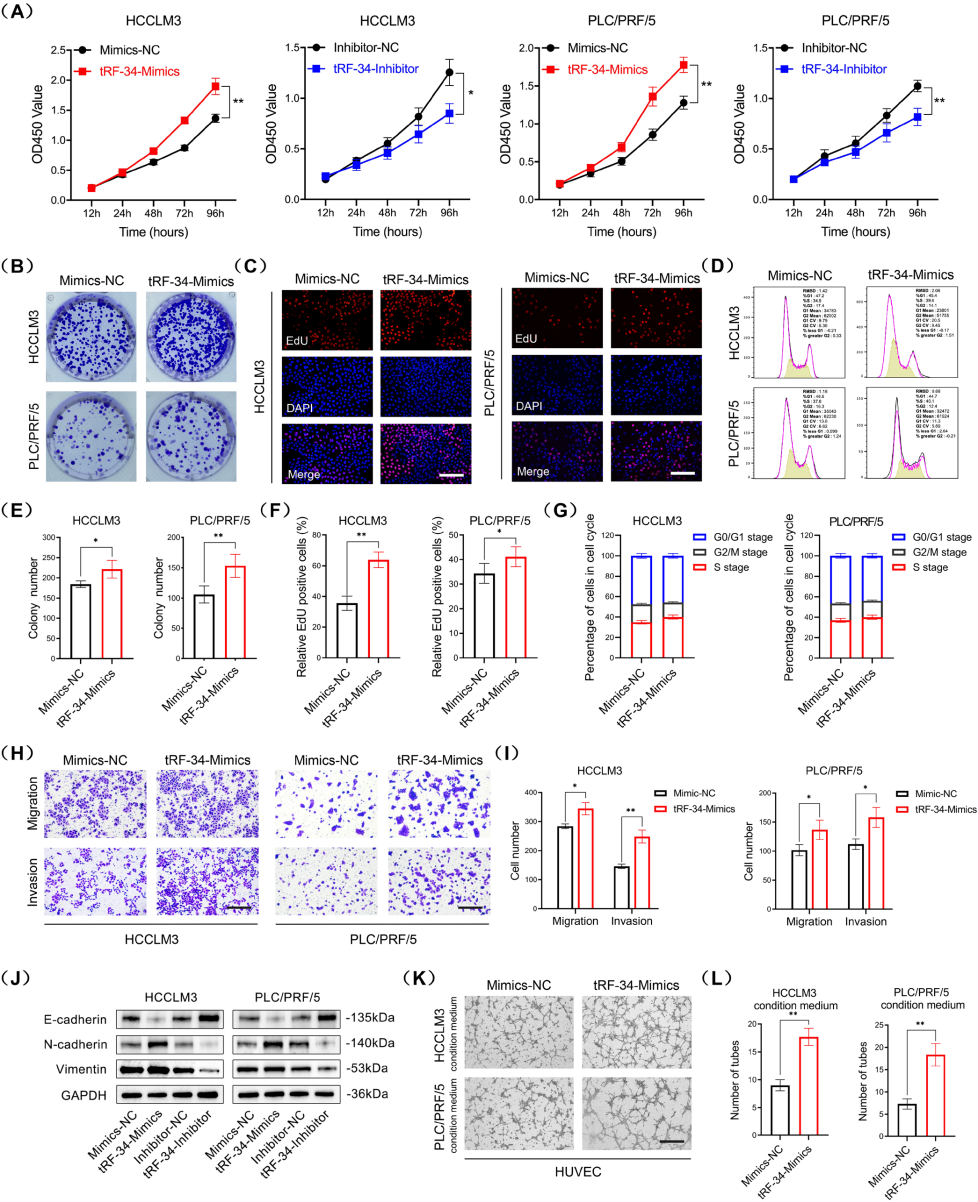
tRF‐34‐P4R8YP9LON4VHM could play an oncogenic role and drive angiogenesis in HCC. (A) The CCK8 assays showed that overexpression of tRF‐34‐P4R8YP9LON4VHM significantly promoted HCC cell proliferation. (B) The colony formation assays showed that overexpression of tRF‐34‐P4R8YP9LON4VHM significantly enhanced HCC cell growth. (C) EdU assays showed that overexpression of tRF‐34‐P4R8YP9LON4VHM increased the proportion of proliferating cells. (D) Cell cycle analysis revealed that overexpression of tRF‐34‐P4R8YP9LON4VHM induced cells to be arrested at the S/G2/M phase. (E) Bar chart of colony formation assays. (F) Bar chart of EdU assays. (G) Stacked chart of cell cycle analysis. (H, I) Transwell assays showed that overexpression of tRF‐34‐P4R8YP9LON4VHM significantly enhanced HCC cell migration and invasion abilities. (J) Western blotting assays showed that tRF‐34‐P4R8YP9LON4VHM promoted HCC cell EMT. (K, L) Tube formation assays showed that conditioned medium collected from HCC cells transfected with tRF‐34‐Mimics could promote HUVECs capillary tube formation ability. Scale bar, 100 μm. All data are shown as the mean ± SEM of 3 independent experiments. *p < 0.05; **p < 0.01; ***p < 0.001.

As shown in Figure 1H, HCCLM3 exhibited the highest expression level of tRF‐34‐P4R8YP9LON4VHM, while PLC/PRF/5 was the least upregulated cell line. According to this result, we chose HCCLM3 and PLC/PRF/5 to investigate the oncogenic role of tRF‐34‐P4R8YP9LON4VHM in vitro. First, we performed transient cell transfection using tRF‐34‐P4R8YP9LON4VHM mimics (tRF‐34‐Mimics) and their negative control (Mimics‐NC), as well as tRF‐34‐P4R8YP9LON4VHM inhibitor and their negative control (Inhibitor‐NC), in both HCCLM3 and PLC/PRF/5 cells. The transient transfection efficiency of tRF‐34‐P4R8YP9LON4VHM mimics and inhibitors was shown in Figure S2A. To examine the impact of tRF‐34‐P4R8YP9LON4VHM on tumour cell growth, CCK8 and colony formation assays revealed that the overexpression of tRF‐34‐P4R8YP9LON4VHM significantly enhanced cell proliferation compared to that in the Mimics‐NC group (Figure 3A,B,E). Conversely, the knockdown of tRF‐34‐P4R8YP9LON4VHM exhibited the opposite effects in comparison with the Inhibitor‐NC group (Figure S2B,C). Similarly, we performed EdU staining assay to evaluate the proportion of proliferating cells and the results demonstrated that tRF‐34‐Mimics led to an increase in the ratio of EdU‐positive cells (Figure 3C,F), whereas tRF‐34‐Inhibition reversed this effect (Figure S2D,E). Meanwhile, cell cycle analysis revealed that cells were arrested at the S/G2/M phase upon upregulation of tRF‐34‐P4R8YP9LON4VHM (Figure 3D,G), and tRF‐34‐Inhibitor resulted in more cells at the G0/G1 phase compared to the Inhibitor‐NC group (Figure S2F,H). Furthermore, we conducted Transwell assays to investigate the potential involvement of tRF‐34‐P4R8YP9LON4VHM in cell migration and invasion abilities. The results showed that more cells could migrate into the lower chamber of Transwell inserts after being transfected with tRF‐34‐Mimics, suggesting that the overexpression of tRF‐34‐P4R8YP9LON4VHM might be able to promote the migratory ability of HCC cells. Similarly, after pre‐layering of Matrigel on the upper chamber, the number of cells on the underside of the inserts was increased, suggesting that it might promote the cell invasion ability (Figure 3H,I). On the flip side, the knockdown of tRF‐34‐P4R8YP9LON4VHM might inhibit the migration and invasive ability of HCC cells (Figure S2G,I). Since EMT is a key mechanism involved in cell migration and invasion, we next investigated the impact of tRF‐34‐P4R8YP9LON4VHM on EMT progression. The results of western blot in both HCCLM3 and PLC/PRF/5 cells showed that the overexpression of tRF‐34‐P4R8YP9LON4VHM could increase the expression levels of mesenchymal markers (N‐cadherin and Vimentin), while a reduction was noted in the epithelial marker (E‐cadherin) (Figure 3J). As mentioned above, the overexpression of tRF‐34‐P4R8YP9LON4VHM was positively correlated with high MVD in HCC tissue microarrays. Therefore, we conducted the tube formation assay to evaluate the effect of tRF‐34‐P4R8YP9LON4VHM expression on angiogenesis and the results showed that the conditioned medium collected from HCC cells transfected with tRF‐34‐Mimics could promote HUVEC capillary tube formation ability (Figure 3K,L), whereas the conditioned medium collected from HCC cells transfected with tRF‐34‐Inhibitor reduced the amounts of capillary tube formation (Figure S4J,K). However, the result of the cell apoptosis assay did not show significant positivity after the transfection (Figure S3). Taken together, these results suggested that tRF‐34‐P4R8YP9LON4VHM could play an oncogenic role and drive angiogenesis in the carcinogenesis of HCC.

### 3.5 tRF‐34‐P4R8YP9LON4VHM Reduces DAB2IP Expression by Directly Targeting Its 3'‐UTR

**FIGURE 4:**
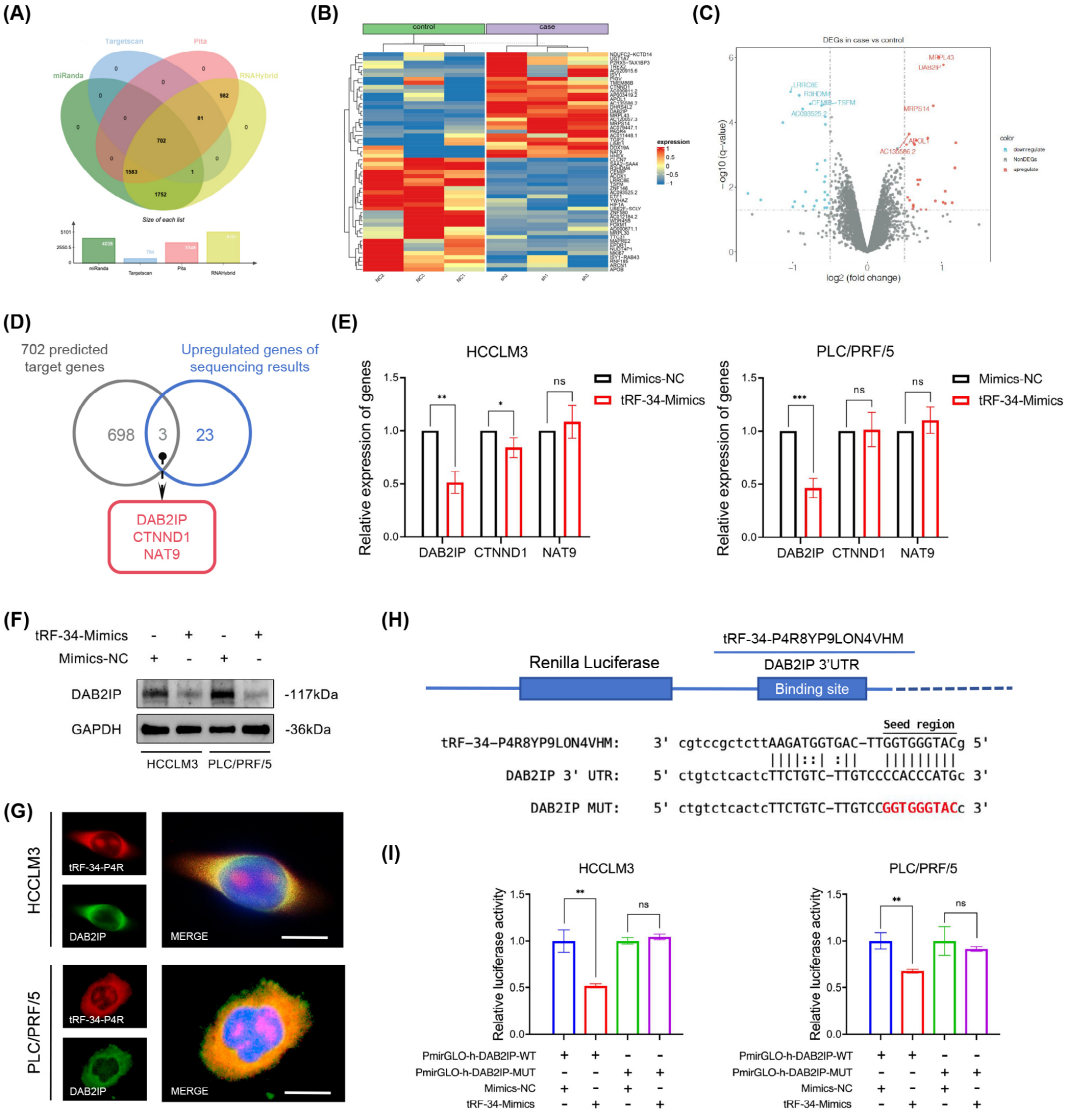
tRF‐34‐P4R8YP9LON4VHM reduces DAB2IP expression by directly targeting its 3′‐UTR. (A) Venn diagram of tRF‐34‐P4R8YP9LON4VHM target gene prediction. (B) Heatmap of mRNA sequencing. (C) Volcano plot of mRNA sequencing. (D) A total of three genes were selected from screening between tRF‐34‐P4R8YP9LON4VHM predicted target genes and upregulated genes of sequencing. (E) mRNA expression of three potential target genes in HCCLM3 and PLC/PRF/5 cells transfected with tRF‐34‐mimics and Mimics‐NC. (F) Protein levels of DAB2IP in HCCLM3 and PLC/PRF/5 cells transfected with tRF‐34‐mimics and Mimics‐NC. (G) The localization of tRF‐34‐P4R8YP9LON4VHM (red) and DAB2IP (green) in HCCLM3 and PLC/PRF/5 cells using FISH‐IF assay. Scale bar, 10 μm. (H) 3′‐UTR fragment of wide‐type (WT) and mutated (MUT) which disrupted interaction with tRF‐34‐P4R8YP9LON4VHM. (I) The wild type or mutated reporter plasmid was co‐transfected into HCCLM3 and PLC/PRF/5 cells with tRF‐34‐mimics or Mimics‐NC. All data are shown as the mean ± SEM of 3 independent experiments. *p < 0.05; **p < 0.01; ***p < 0.001.

tsRNAs could participate in regulating mRNA stability analogous to miRNAs in a canonical or non‐canonical way [19]. Therefore, to explore the downstream molecular function of tRF‐34‐P4R8YP9LON4VHM, we employed multiple bioinformatics tools, including miRanda, TargetScan, Pita, and RNAhybrid, to predict potential target genes (Figure 4A). In order to narrow down the range of these potential target genes, we conducted the mRNA sequencing on HCCLM3 cells transfected with tRF‐34‐Inhibitor and Inhibitor‐NC. The heatmap and volcano plot showed that there were 26 significantly upregulated genes (Figure 4B,C). The overlapping result between the predicted target genes and upregulated genes after tRF‐34‐Inhibit transfection identified 3 potential target genes (DAB2IP, CTNND1, and NAT9) (Figure 4D). To select a gene with the highest likelihood of binding to the 3′UTR of tRF‐34‐P4R8YP9LON4VHM, tRF‐34‐Mimics were transfected into HCCLM3 and PLC/PRF/5 cells, and after 48 h, the mRNA expression in the cells was analysed using qRT‐PCR. We found that the expression levels of DAB2IP mRNA were decreased in both HCCLM3 and PLC/PRF/5 cells after overexpressing tRF‐34‐P4R8YP9LON4VHM (Figure 4E). Meanwhile, the protein levels of DAB2IP in the presence of tRF‐34‐Mimics were detected. The western blot results revealed that tRF‐34‐Mimics significantly decreased the protein levels of DAB2IP in HCC cell lines (Figure 4F). The tumour‐suppressor DAB2IP (Disabled homologue 2 interacting protein) is a RasGAP and negatively controls Ras‐dependent mitogenic signals. FISH‐IF assay proved that both tRF‐34‐P4R8YP9LON4VHM and DAB2IP were mainly located in the cytoplasm of HCCLM3 and PLC/PRF/5 cells (Figure 4G). Target prediction tool indicated that there were predicted specific targets for tRF‐34‐P4R8YP9LON4VHM in the seed regions of the DAB2IP 3′‐UTR (Figure 4H). The empty vector, reporter vector PmirGLO carrying the DAB2IP 3′‐UTR, and the DAB2IP 3′‐UTR mutant binding site were co‐transfected with tRF‐34‐Mimics or Mimics‐NC into HCCLM3 and PLC/PRF/5 cells. After 48 h, the cells were collected for luciferase detection. As expected, tRF‐34‐Mimics significantly reduced the relative luciferase activity when co‐transfected with the pmirGLO‐h‐DAB2IP‐WT compared with PmirGLO‐h‐DAB2IP‐MUT in both HCCLM3 and PLC/PRF/5 cell lines (Figure 4I). Through the above experiments, we concluded that tRF‐34‐P4R8YP9LON4VHM could promote the progression of HCC by directly targeting DAB2IP 3′‐UTR and silencing its expression.

### 3.6 tRF‐34‐P4R8YP9LON4VHM Mimicry Effects on HCC Cells Were Partially Abolished by Overexpression of DAB2IP

**FIGURE 5:**
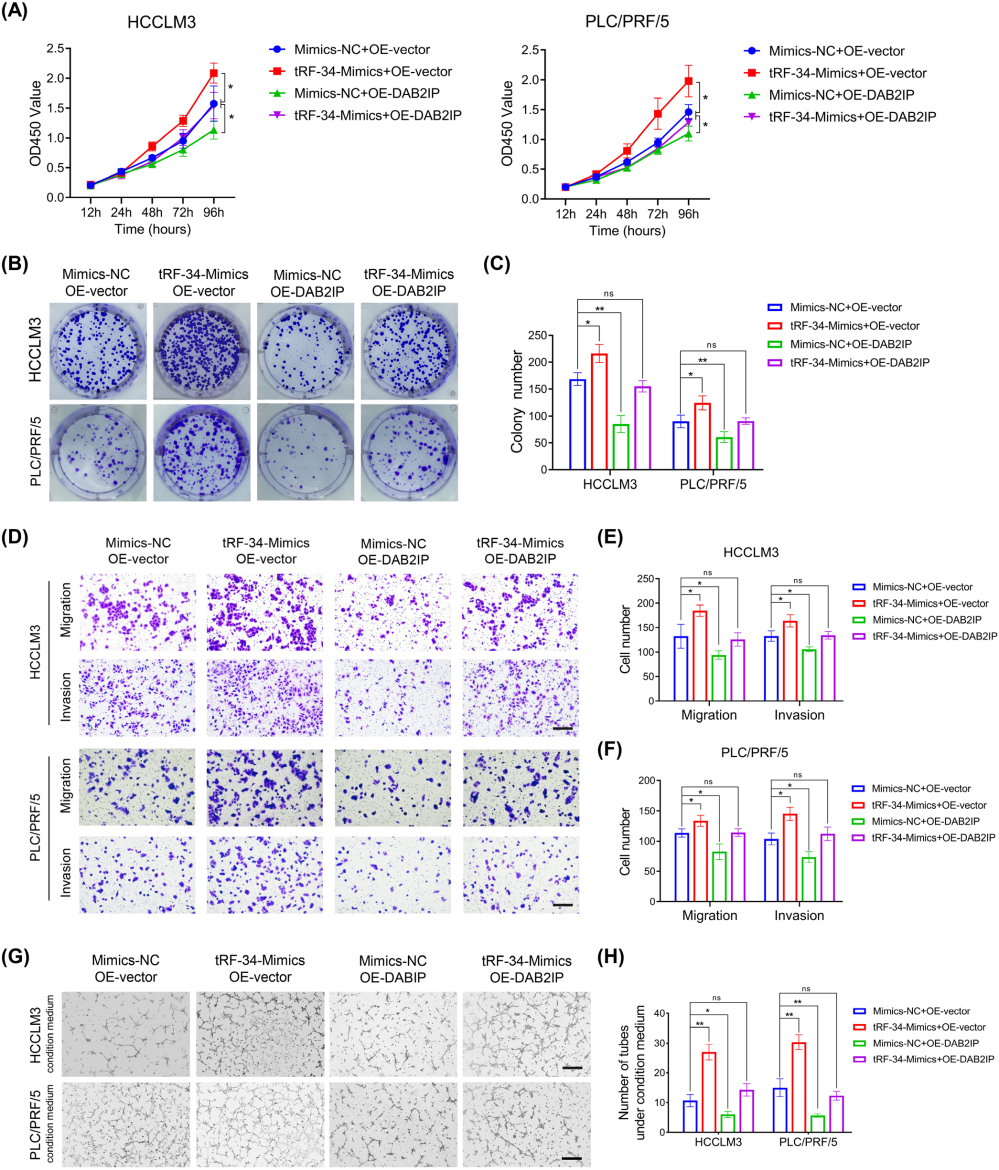
Rescue experiment was performed for confirming the relationship between tRF‐34‐P4R8YP9LON4VHM and DAB2IP. (A) Cell viability of HCCLM3 and PLC/PRF/5 cells after co‐transfected with tRF‐34‐Mimics and OE‐DAB2IP was detected by CCK‐8 assay. (B) and (C) Colony formation assays in the HCCLM3 and PLC/PRF/5 cells after co‐transfected with tRF‐34‐Mimics and OE‐DAB2IP. (D–F) Representative images and bar graphs were depicted to investigate the migration and invasion ability of HCC cells after the 24 h co‐transfection. (G, H) Representative images and bar graphs were depicted to investigate the capillary tube formation ability of HUVECs cells under conditioned medium. Scale bar, 100 μm. All data are shown as the mean ± SEM of 3 independent experiments. *p < 0.05; **p < 0.01; ***p < 0.001.

First, we constructed stable HCC cell lines overexpressing DAB2IP by lentiviral transduction of the HCCLM3 and PLC/PRF/5 cell lines and verified the overexpression efficiency at the mRNA and protein level (Figure S4). Then, HCCLM3 and PLC/PRF/5 cells transfected with OE‐DAB2IP were co‐transfected with tRF‐34‐Mimics and Mimics‐NC, and we performed the rescue assays to explore the regulatory mechanism of the tRF‐34‐P4R8YP9LON4VHM/DAB2IP axis in HCC. As a result, we found that OE‐DAB2IP could significantly suppress cell growth through CCK8 and colony formation assays, and the effect of tRF‐34‐Mimics on proliferation ability was reversed by DAB2IP overexpression (Figure 5A–C). In addition, the decreased migration and invasion ability of HCC cells via DAB2IP overexpression was recovered after the co‐transfection of tRF‐34‐Mimics (Figure 5D–F). Moreover, conditioned medium collected from OE‐DAB2IP HCC cells could impair HUVECs capillary tube formation ability, while the pro‐angiogenic effect of tRF‐34‐Mimics conditioned medium could be reversed by OE‐DAB2IP conditioned medium, supporting that DAB2IP was involved in tRF‐34‐P4R8YP9LON4VHM‐mediated angiogenesis (Figure 5G,H). Taken together, the above results suggested that tRF‐34‐P4R8YP9LON4VHM contributed to tumour progression and angiogenesis via silencing DAB2IP expression in HCC cells.

### 3.7 tRF‐34‐P4R8YP9LON4VHM Regulates the MEK/ERK Pathway by Silencing DAB2IP Expression in HCC Cells and Drives Tumour Cell‐Induced Angiogenesis by Secreting VEGFA

**FIGURE 6:**
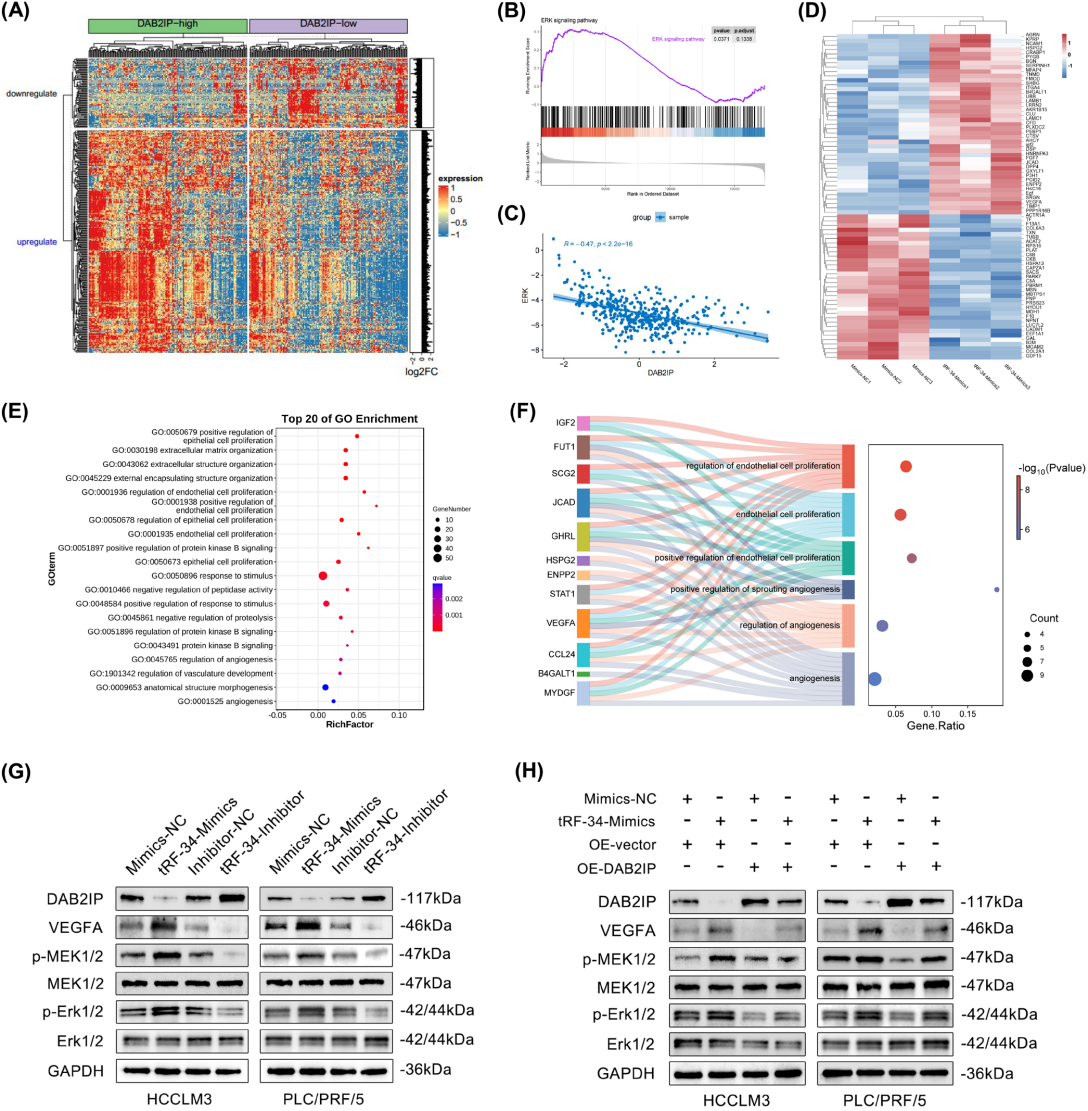
tRF‐34‐P4R8YP9LON4VHM regulates the MEK/ERK pathway in HCC cells and drives tumour cell‐induced angiogenesis. (A) Heatmap of the differentially expressed mRNAs between the DAB2IP‐high and DAB2IP‐low groups based on the median expression from TCGA. (B) GSEA analysis of the MEK/ERK signalling pathway. (C) Correlation analysis between DAB2IP and ERK. (D) Heatmap of proteome difference analysis showed a total of 41 proteins with increased expression in the tRF‐34‐Mimics culture supernatant. (E) GO enrichment analysis of differentially expressed proteins in the tRF‐34‐Mimics culture supernatant. (F) Sankey dot enrichment revealed angiogenesis‐related proteins including VEGFA. (G) Western blot analysis of DAB2IP, VEGFA, p‐MEK1/2, MEK1/2, p‐ERK1/2, and ERK1/2 in HCC cells transfected with tRF‐34‐Mimics and tRF‐34‐Inhibitor. (H) Western blot analysis of DAB2IP, VEGFA, p‐MEK1/2, MEK1/2, p‐ERK1/2, and ERK1/2 in HCC cells co‐transfected with tRF‐34‐Mimics and OE‐DAB2IP. GAPDH was used as a loading control. All data are shown as the mean ± SEM of 3 independent experiments. *p < 0.05; **p < 0.01; ***p < 0.001.

Previous studies have reported that the downregulation of DAB2IP can activate the MEK/ERK signalling pathway, promoting the malignant progression of colorectal cancer [20]. Therefore, we used bioinformatics analysis to further determine whether tRF‐34‐P4R8YP9LON4VHM could regulate the MEK/ERK signalling pathway mediated by DAB2IP in HCC. First, we obtained the raw count expression profile data of HCC from the TCGA database. After FPKM standardisation, we divided the overall samples into DAB2IP‐high and DAB2IP‐low groups based on the median expression, and the heatmap of the differentially expressed mRNAs between the two groups was shown in Figure 6A. Through GSEA and correlation analysis, we found that DAB2IP is negatively correlated with the MEK/ERK signalling pathway in HCC (Figure 6B,C). Aside from promoting the proliferation, migration, and invasion abilities of HCC cells by silencing the expression of the target gene DAB2IP, we assumed that tRF‐34‐P4R8YP9LON4VHM could accelerate HUVECs tube formation ability by secreting growth factors or pro‐angiogenic products from HCC cells into the extracellular environment. Therefore, we collected the culture supernatants from HCCLM3 cells transfected with tRF‐34‐Mimics and Mimics‐NC for proteome difference analysis. The heatmap revealed a total of 74 significantly differentially expressed proteins, and there were 41 proteins with increased expression in the tRF‐34‐Mimics culture supernatant (Figure 6D). GO enrichment was shown in Figure 6E, and sankey dot enrichment revealed angiogenesis‐related proteins including VEGFA (Figure 6F). Given that VEGFA acted as an inducer of angiogenesis and the activation of the MEK/ERK signalling pathway could induce VEGFA expression, we assumed whether tRF‐34‐P4R8YP9LON4VHM could promote angiogenesis via the DAB2IP/MEK/ERK/VEGFA axis [21]. Upon overexpression of tRF‐34‐P4R8YP9LON4VHM, the protein expression of DAB2IP was reduced, while the expression of VEGFA and the phosphorylation levels of MEK/ERK were increased. Conversely, when tRF‐34‐Inhibitor was transfected into cells, the expression of DAB2IP increased, and the expression of VEGFA and the phosphorylation levels of MEK/ERK decreased (Figure 6G). The aforementioned expression changes were also correspondingly validated in the rescue experiment, where DAB2IP overexpression could counteract the effects of tRF‐34‐Mimics on the expression of VEGFA and the phosphorylation levels of MEK/ERK (Figure 6H). Based on these results, we inferred that tRF‐34‐P4R8YP9LON4VHM could activate MEK/ERK phosphorylation and participate in the modulation of VEGFA expression changes by regulating DAB2IP expression, thereby promoting the malignant biological processes of HCC.

### 3.8 Targeting tRF‐34‐P4R8YP9LON4VHM Suppresses HCC Cell Growth In Vivo

**FIGURE 7:**
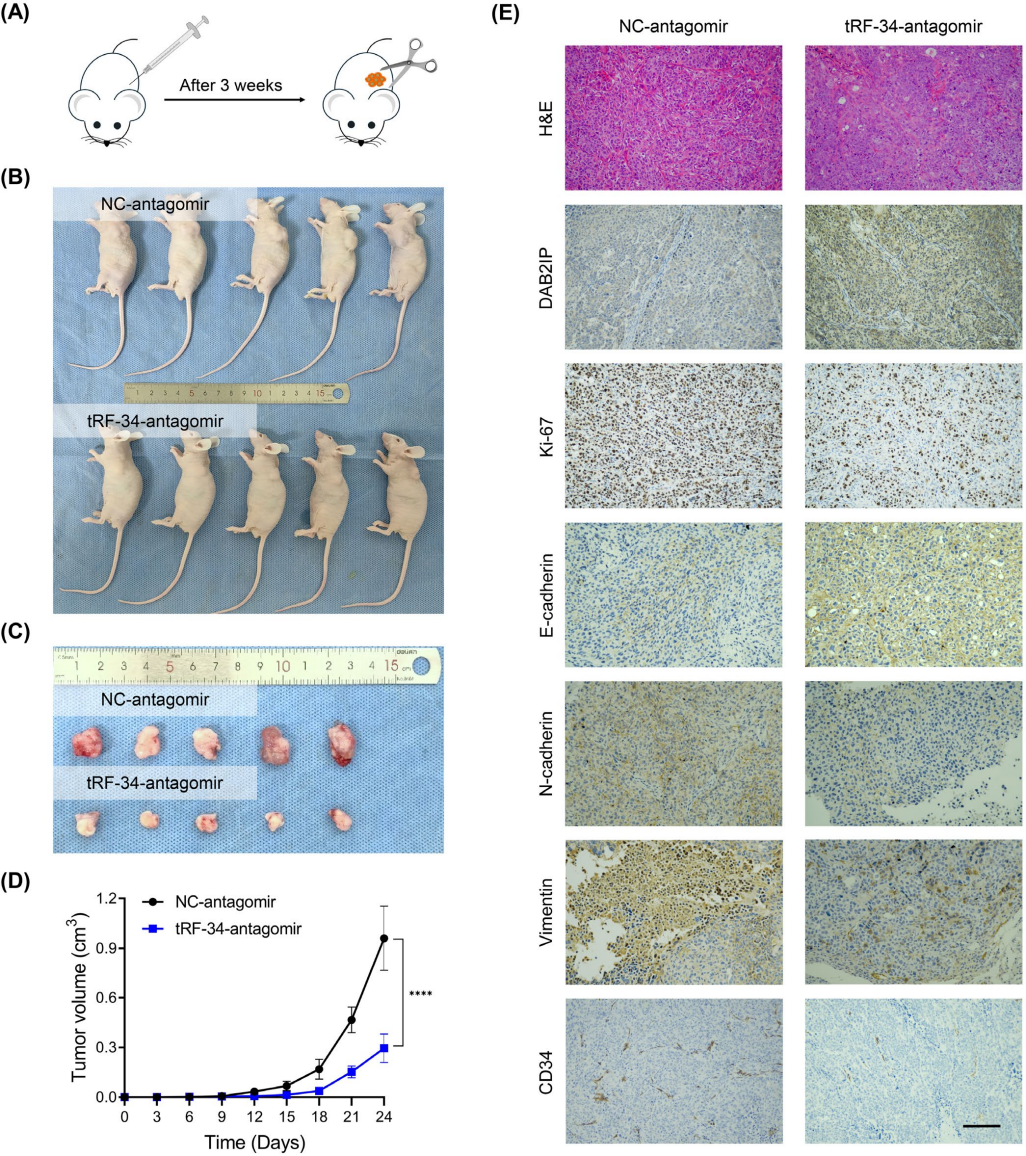
Downregulation of tRF‐34‐P4R8YP9LON4VHM suppresses HCC cell growth in vivo. (A) The cartoon illustrating the animal model. (B) Xenograft mice image. (C) Dissected tumours from nude mice treated with tRF‐34‐antagomir and NC‐antagomir. (D) Corresponding volumes of tumours in xenograft mice were measured. (E) Representative images of H&E, DAB2IP, Ki‐67, E‐cadherin, N‐cadherin, Vimentin, and CD34 were shown. Scale bar, 100 μm. All data are shown as the mean ± SEM of 3 independent experiments. *p < 0.05; **p < 0.01; ***p < 0.001.

Considering that tRF‐34‐P4R8YP9LON4VHM acted as an oncogenic role in HCC, we hypothesised that inhibition of tRF‐34‐P4R8YP9LON4VHM might have a therapeutic effect on HCC. To test this hypothesis, we synthesised tRF‐34‐antagomir for in vivo study (Figure 7A). The results indicated that tumour volume was significantly smaller in the tRF‐34‐antagomir group than that in the NC‐antagomir group during the entire experimental period (Figure 7B–D). Furthermore, the IHC test indicated that administration of tRF‐34‐antagomir also suppressed the expression levels of Ki‐67, N‐cadherin, Vimentin, and CD34 protein levels and increased the expression levels of DAB2IP and E‐cadherin in the xenograft tumour tissues (Figure 7E). Thus, these findings suggested that the inhibition of tRF‐34‐P4R8YP9LON4VHM could suppress HCC tumour growth in vivo.

## 4 Discussion

Hepatocellular carcinoma (HCC) is the most prevalent form of liver cancer, frequently linked to chronic liver conditions such as hepatitis B and C infections, along with cirrhosis [22]. The biological processes underlying HCC are intricate and involve various factors including abnormal activation of molecular signalling pathways, differentiation of HCC cells, and regulation of angiogenesis [23]. The development and popularisation of next‐generation sequencing technology could enhance our understanding of the molecular mechanisms of tumorigenesis and pave the way for new discoveries related to non‐coding RNAs [24]. Recent evidence suggests that tsRNAs were dysregulated in cancer and played a critical role in the pathogenic processes of various cancers, including pancreatic cancer [25], breast cancer [26], non‐small cell lung cancer [27], and gastric cancer [28]. Also, many studies have reported that tsRNAs were dysregulated in HCC. tRF‐Gln‐TTG‐006, which was remarkably upregulated in HCC serum, was released from tumour cells and served as a novel biomarker of HCC [29]. Weilin Wang et al. demonstrated that 5'‐tRF‐Gly is a novel tumour‐promoting factor that could serve as a potential diagnostic biomarker or therapeutic target for HCC [30]. Although some studies of tsRNAs in HCC have been reported, the function and clinical significance of most tsRNAs in HCC angiogenesis remained largely unknown and their specific mechanisms had not been elucidated.

Importantly, we identified tRF‐34‐P4R8YP9LON4VHM, a 5'‐tiRNA‐Gly‐GCC that was elevated in HCC tissues. The clinical results showed that high expression levels of tRF‐34‐P4R8YP9LON4VHM were significantly correlated with tumour progression and angiogenesis. Thus, we performed gain‐ and loss‐of‐function experiments in HCC cells in vitro, which showed that tRF‐34‐P4R8YP9LON4VHM could promote proliferation, migration, invasion, and EMT of HCC cells, and enhanced tube formation ability of HUVECs under HCC cells conditional culture medium.

We further uncovered the mechanisms by which tRF‐34‐P4R8YP9LON4VHM contributes to HCC occurrence and development. Current studies have shown that some tsRNAs could bind directly to the 3'‐UTR of target mRNAs to trigger either translational repression or destabilisation of the mRNA, analogous to a miRNA‐like mechanism [31, 32]. Thus, we used bioinformatic tools and mRNA sequencing to screen out the target gene of tRF‐34‐P4R8YP9LON4VHM. DAB2IP, a Ras GTPase‐activating protein, functions as a tumour suppressor and is involved in various cellular processes, including cell growth, migration, and signal transduction. It has also been reported that DAB2IP is functionally involved in angiogenesis in clear cell renal cell carcinoma [33]. In our study, we found that upregulation of tRF‐34‐P4R8YP9LON4VHM could significantly decrease the level of DAB2IP mRNA as well as the protein level. Then, we verified that tRF‐34‐P4R8YP9LON4VHM could bind to specific sequences in the 3'‐UTR of DAB2IP via a dual luciferase assay. Moreover, overexpression of DAB2IP inhibited HCC cell aggressiveness similar to the effects of tRF‐34‐P4R8YP9LON4VHM downregulation. More importantly, in our rescue experiments, we demonstrated that the overexpression of DAB2IP significantly mitigated the effects of the tRF‐34‐P4R8YP9LON4VHM mimics on the HCC cells proliferation, migration, invasion, and tumour cell‐induced angiogenic properties. This finding suggested that DAB2IP played a crucial role in counteracting the oncogenic influences of tRF‐34‐P4R8YP9LON4VHM, highlighting its potential as a regulatory factor in the progression of HCC. By partially abolishing these mimic effects, our results not only underscored the importance of DAB2IP in modulating cancer cell behaviour but also pointed to potential therapeutic strategies that could target this pathway to inhibit tumour growth and spread.

The downstream molecular signalling involved in the regulation of tRF‐34‐P4R8YP9LON4VHM/DAB2IP axis on HCC progression still remains unknown. A previous study showed that efficient downregulation of DAB2IP could activate the MEK/ERK signalling pathway in colorectal cancer [20]. In our study, we speculated that tRF‐34‐P4R8YP9LON4VHM might contribute to HCC progression via the DAB2IP/MEK/ERK axis. Therefore, we first analysed the raw data of HCC from the TCGA database and found that DAB2IP is negatively correlated with the MEK/ERK signalling pathway in HCC. HCC growth often requires angiogenesis which is regulated by factors like VEGFA, which promotes blood supply to the tumour and facilitate its growth and metastasis [3]. Thus, we employed proteome difference analysis to systematically investigate and characterise the composition of the supernatants, which showed that VEGFA was upregulated in the culture supernatants from HCCLM3 cells transfected with tRF‐34‐Mimics. In addition, some studies have reported that MEK/ERK signalling pathway may be partially responsible for the accumulation of VEGFA and leads to tumour cell‐mediated angiogenesis [34, 35]. Besides, VEGFA could activate several angiogenic signalling pathways in HUVECs, which ultimately affect HUVECs proliferation, migration, invasion, and tube formation capacity [21, 36, 37, 38]. Our results of western blots indicated that tRF‐34‐P4R8YP9LON4VHM had the potential to activate the MEK/ERK signalling pathway and enhance the secretion of VEGFA from HCC cells. This effect appeared to occur through the downregulation of DAB2IP expression, suggesting a mechanism by which tRF‐34‐P4R8YP9LON4VHM contributed to the tumorigenic processes associated with HCC. Additionally, when the level of DAB2IP was overexpressed, we observed a reversal of the positive effects induced by tRF‐34‐Mimics on both the activation of the MEK/ERK signalling pathway and VEGFA secretion. This finding highlighted the significance of DAB2IP as a potential regulatory factor that could counteract the oncogenic actions of tRF‐34‐P4R8YP9LON4VHM, further elucidating the complex interplay between these molecular players in HCC.

In summary, this paper provided sufficient evidence that tRF‐34‐P4R8YP9LON4VHM was significantly upregulated in HCC tissues and it could promote proliferation, migration, invasion of HCC cells and enhance tumour cell‐induced angiogenesis. Mechanistically, we identified that tRF‐34‐P4R8YP9LON4VHM could bind directly to the 3'‐UTR of DAB2IP and silence its expression, thus activating the MEK/ERK signalling pathway and promoting the secretion of VEGFA. These findings presented a promising new potential therapeutic target for the treatment of HCC and a new explanation for the occurrence and development of HCC.

## Author Contributions

**Tianxin Xu:** conceptualization (equal), methodology (equal), software (equal), writing – original draft (equal). **Han Hua:** conceptualization (equal), methodology (equal), software (equal). **Fei Song:** data curation (equal). **Nannan Zhang:** investigation (equal), visualization (equal). **Cheng Gao:** data curation (equal). **Zhong Chen:** conceptualization (equal), writing – review and editing (equal).

## Ethics Statement

This study was performed in line with the principles of the World Medical Association Declaration of Helsinki (as revised in 2013). Approval was granted by the Ethics Committee of the Affiliated Hospital of Nantong University (ethical review report number: 2022‐L062). Before collecting samples, written informed consent was obtained from all individual participants included in the study and data were analysed anonymously. All animal experiments were performed in accordance with a protocol approved by the Animal Experimental Ethical Committee of Nantong University, China (ethical review report number: S20220228‐004).

## Conflicts of Interest

The authors declare no conflicts of interest.

## Supporting information


**Figure S1.** Characteristics of tRF‐34‐P4R8YP9LON4VHM. (A) Based on the UCSC Genome Browser database, tRF‐34‐P4R8YP9LON4VHM is located on chromosome chr1 (q23.3) with coordinates of 161,413,094 to 161,413,127. (B) The brief introduction of tRF‐34‐P4R8YP9LON4VHM in the MINTbase v2.0. (C) tRF‐34‐P4R8YP9LON4VHM was derived from of tRNA‐Gly‐GCC with the length of 34 nt and the cleavage site was pointed with the red scissor symbols. (D) Sanger sequencing of confirmed that the qRT‐PCR product contained the full‐length sequence of tRF‐34‐P4R8YP9LON4VHM. (E) Nuclear‐cytoplasmic separation assay assessed the intracellular localization of tRF‐34‐P4R8YP9LON4VHM in HCCLM3 and PLC/PRF/5 cells. All data are shown as the mean ± SEM of 3 independent experiments. *p < 0.05; **p < 0.01; ***p < 0.001.

**Figure S2.** (A) The transient transfection efficiency of tRF‐34‐P4R8YP9LON4VHM mimics and inhibitors. (B, C) The colony formation assays showed that downregulation tRF‐34‐P4R8YP9LON4VHM significantly suppressed HCC cells growth. (D, E) EdU assays showed that downregulation of tRF‐34‐P4R8YP9LON4VHM decreased the proportion of proliferating cells. (F) Cell cycle analysis revealed that downregulation of tRF‐34‐P4R8YP9LON4VHM induced cells to be arrested at the G0/G1 phase. (G) Transwell assays showed that downregulation of tRF‐34‐P4R8YP9LON4VHM significantly suppressed HCC cells migration and invasion abilities. (H) Stacked chart of cell cycle analysis. (I) Bar chart of transwell assays. (J, K) Tube formation assays showed that conditioned medium collected from HCC cells transfected with tRF‐34‐inhibitor could suppress HUVECs capillary tube formation ability. Scale bar, 100 μm. All data are shown as the mean ± SEM of 3 independent experiments. *p < 0.05; **p < 0.01; ***p < 0.001.

**Figure S3.** Cell apoptosis assay did not show the significant positivity after transfection. All data are shown as the mean ± SEM of 3 independent experiments. *p < 0.05; **p < 0.01; ***p < 0.001.

**Figure S4.** Construction of stable HCC cell lines overexpressing DAB2IP by lentiviral transduction of the HCCLM3 and PLC/PRF/5 cell lines and verification of overexpression efficiency at the mRNA and protein level. All data are shown as the mean ± SEM of 3 independent experiments. *p < 0.05; **p < 0.01; ***p < 0.001.

## Data Availability

The data that support the findings of this study are available from the corresponding author upon reasonable request.
