# Supplementary figures and images for: tRF‐34‐P4R8YP9LON4VHM Promotes Hepatocellular Carcinoma Progression and Tumour Cell‐Induced Angiogenesis via the MEK/ERK Pathway
Source: J Cell Mol Med. 2025 Apr 22;29(8):e70560. doi: 10.1111/jcmm.70560 (PMC12014515; doi:10.1111/jcmm.70560)

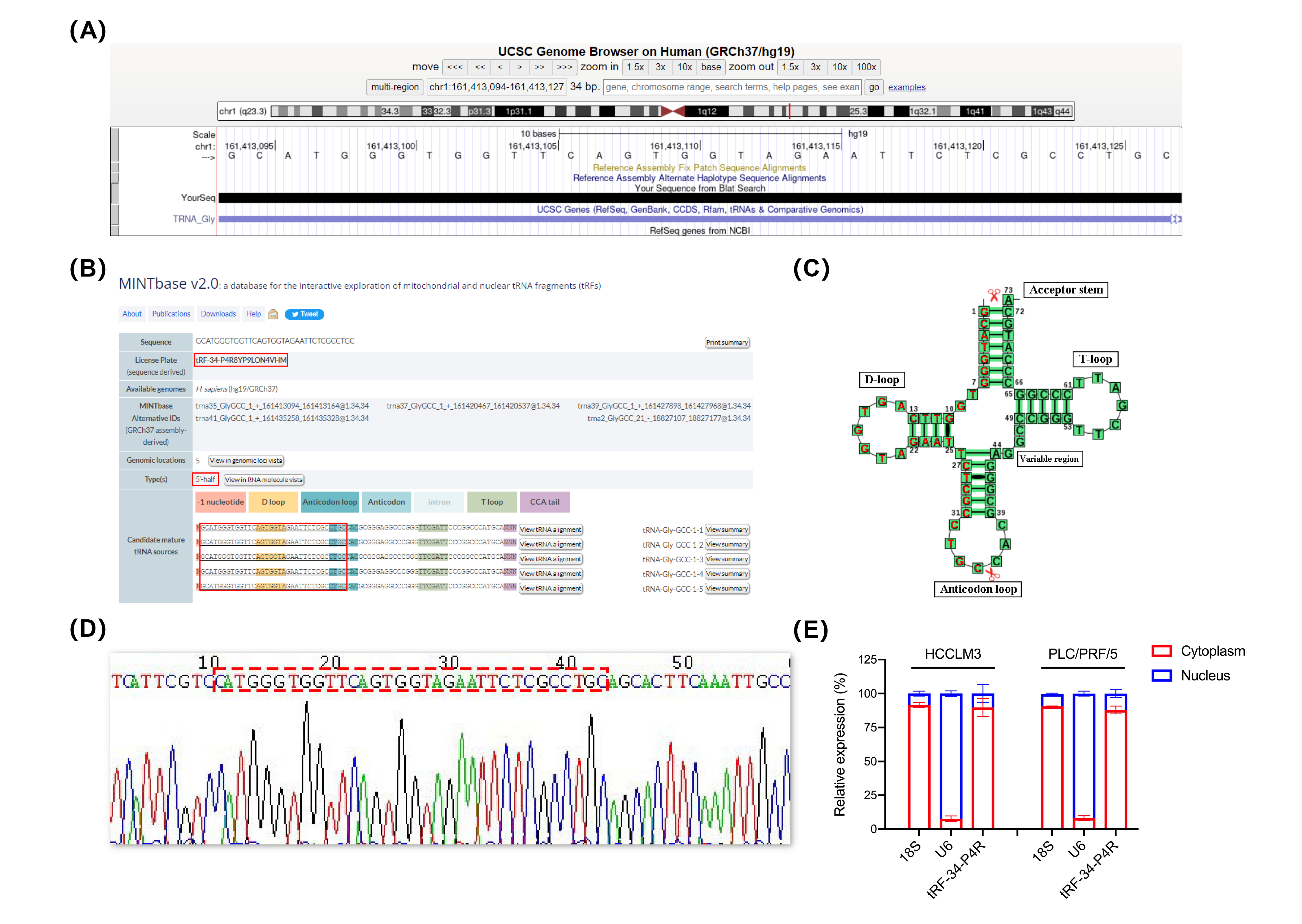

Supplement: Supplementary file 1 — Figure S1. Characteristics of tRF‐34‐P4R8YP9LON4VHM. (A) Based on the UCSC Genome Browser database, tRF‐34‐P4R8YP9LON4VHM is located on chromosome chr1 (q23.3) with coordinates of 161,413,094 to 161,413,127. (B) The brief introduction of tRF‐34‐P4R8YP9LON4VHM in the MINTbase v2.0. (C) tRF‐34‐P4R8YP9LON4VHM was derived from of tRNA‐Gly‐GCC with the length of 34 nt and the cleavage site was pointed with the red scissor symbols. (D) Sanger sequencing of confirmed that the qRT‐PCR product contained the full‐length sequence of tRF‐34‐P4R8YP9LON4VHM. (E) Nuclear‐cytoplasmic separation assay assessed the intracellular localization of tRF‐34‐P4R8YP9LON4VHM in HCCLM3 and PLC/PRF/5 cells. All data are shown as the mean ± SEM of 3 independent experiments. *p < 0.05; **p < 0.01; ***p < 0.001. [file JCMM-29-e70560-s002.jpg]

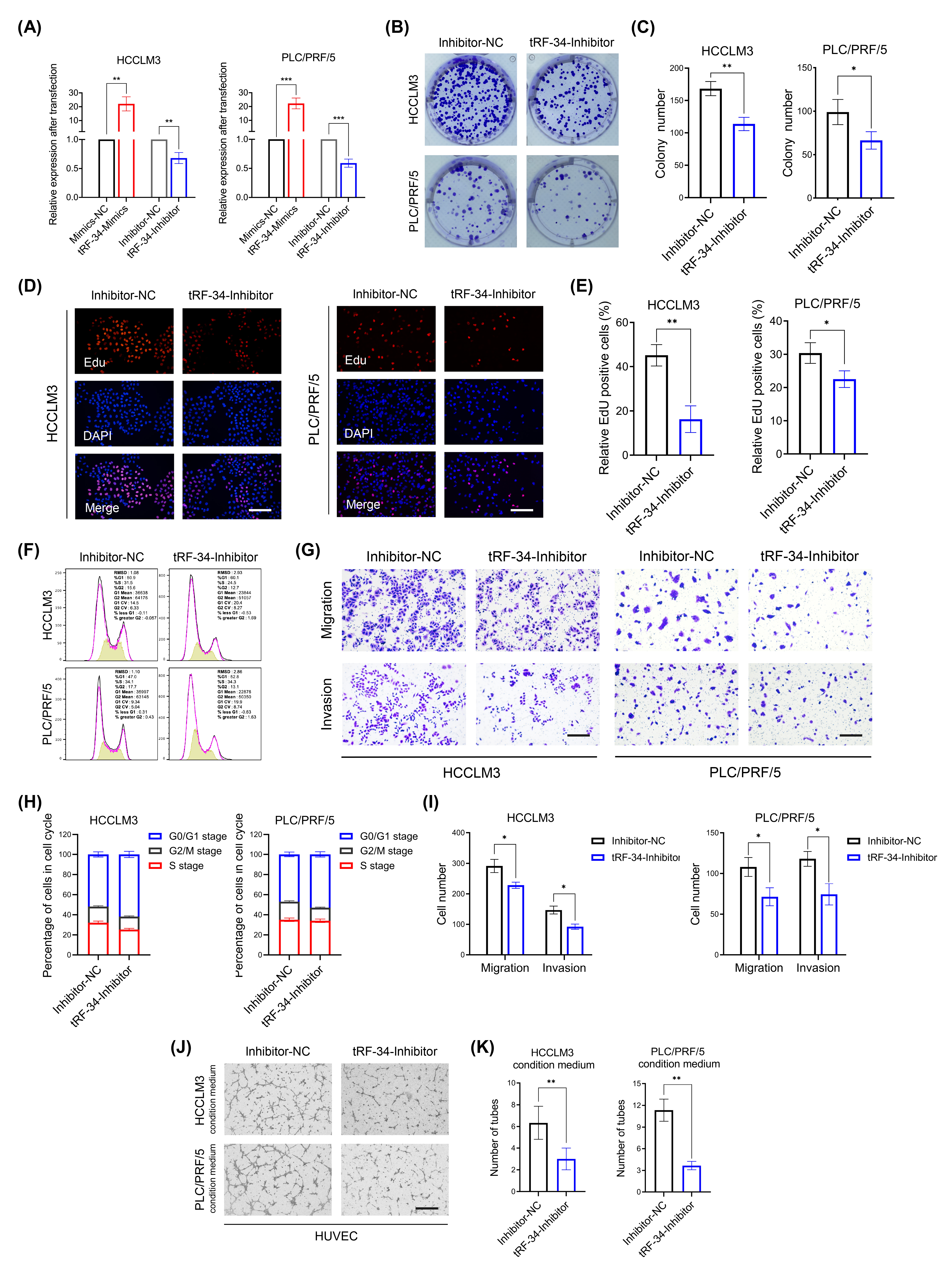

Supplement: Supplementary file 2 — Figure S2. (A) The transient transfection efficiency of tRF‐34‐P4R8YP9LON4VHM mimics and inhibitors. (B, C) The colony formation assays showed that downregulation tRF‐34‐P4R8YP9LON4VHM significantly suppressed HCC cells growth. (D, E) EdU assays showed that downregulation of tRF‐34‐P4R8YP9LON4VHM decreased the proportion of proliferating cells. (F) Cell cycle analysis revealed that downregulation of tRF‐34‐P4R8YP9LON4VHM induced cells to be arrested at the G0/G1 phase. (G) Transwell assays showed that downregulation of tRF‐34‐P4R8YP9LON4VHM significantly suppressed HCC cells migration and invasion abilities. (H) Stacked chart of cell cycle analysis. (I) Bar chart of transwell assays. (J, K) Tube formation assays showed that conditioned medium collected from HCC cells transfected with tRF‐34‐inhibitor could suppress HUVECs capillary tube formation ability. Scale bar, 100 μm. All data are shown as the mean ± SEM of 3 independent experiments. *p < 0.05; **p < 0.01; ***p < 0.001. [file JCMM-29-e70560-s003.jpg]

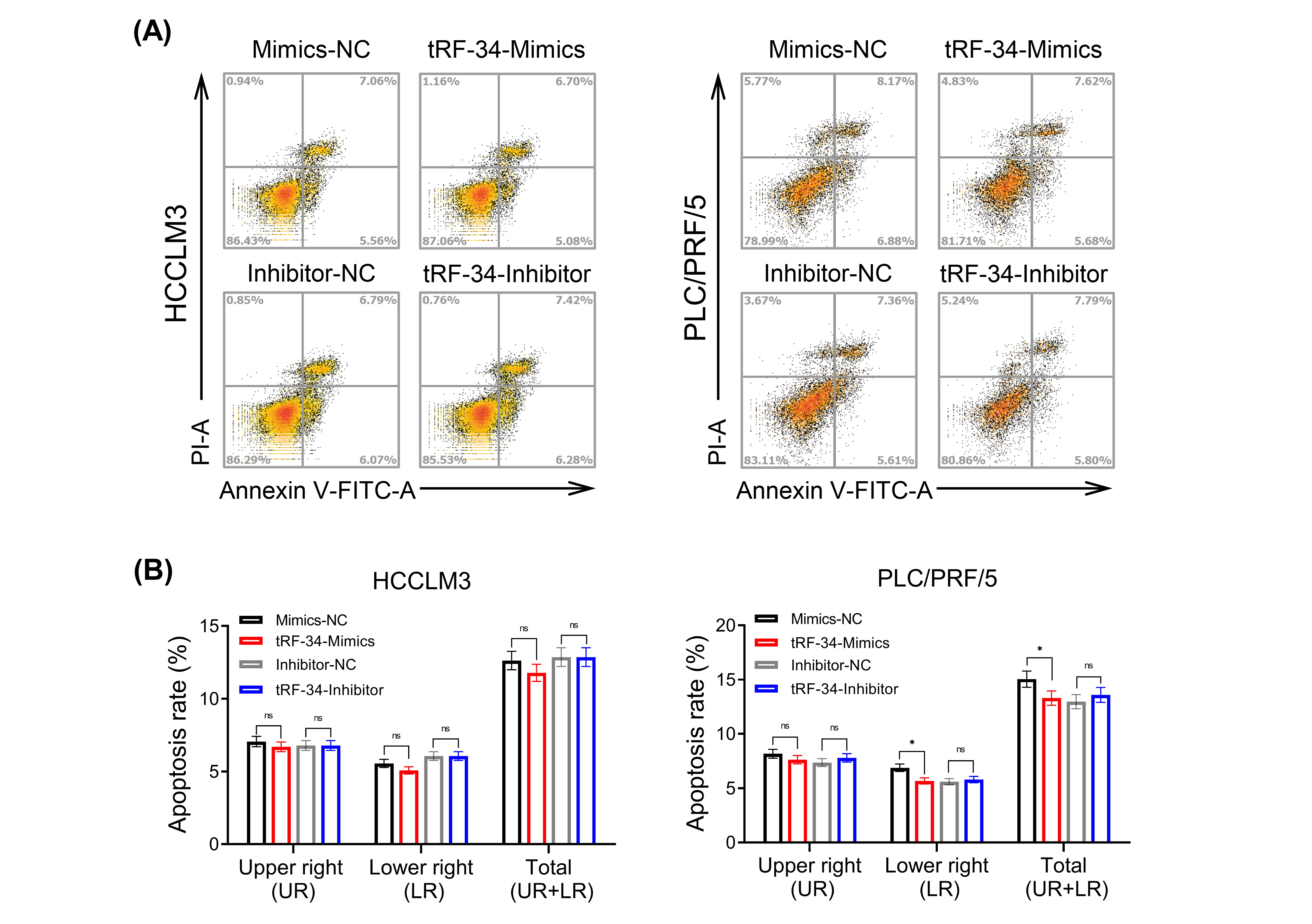

Supplement: Supplementary file 3 — Figure S3. Cell apoptosis assay did not show the significant positivity after transfection. All data are shown as the mean ± SEM of 3 independent experiments. *p < 0.05; **p < 0.01; ***p < 0.001. [file JCMM-29-e70560-s001.jpg]

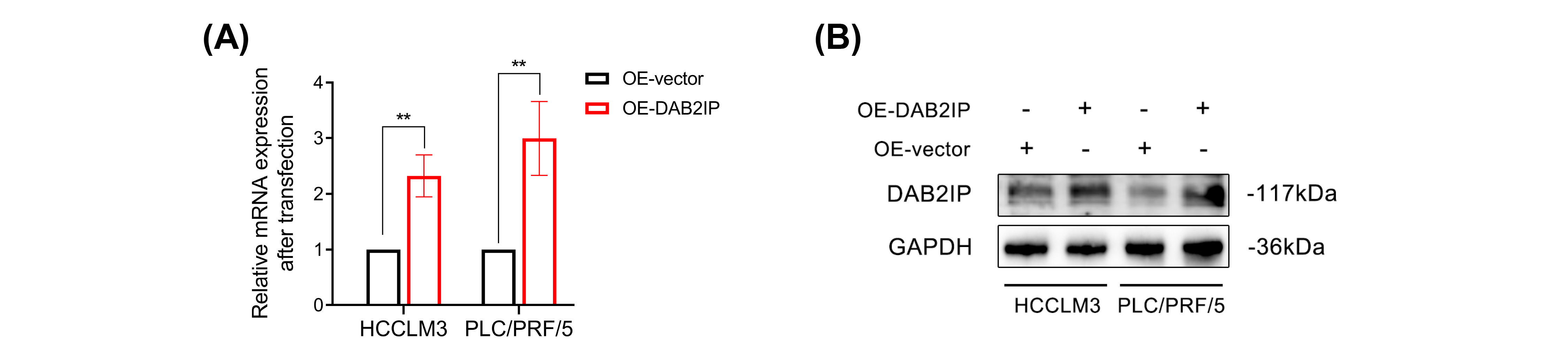

Supplement: Supplementary file 4 — Figure S4. Construction of stable HCC cell lines overexpressing DAB2IP by lentiviral transduction of the HCCLM3 and PLC/PRF/5 cell lines and verification of overexpression efficiency at the mRNA and protein level. All data are shown as the mean ± SEM of 3 independent experiments. *p < 0.05; **p < 0.01; ***p < 0.001. [file JCMM-29-e70560-s004.jpg]
